# Complete Genome Sequence of Noncytopathic Bovine Viral Diarrhea Virus 1 Contaminating a High-Passage RK-13 Cell Line

**DOI:** 10.1128/genomeA.01115-15

**Published:** 2015-10-01

**Authors:** Bora Nam, Ganwu Li, Ying Zheng, Jianqiang Zhang, Kathleen M. Shuck, Peter J. Timoney, Udeni B. R. Balasuriya

**Affiliations:** a Department of Veterinary Science, Maxwell H. Gluck Equine Research Center, University of Kentucky, Lexington, Kentucky, USA; b Department of Veterinary Diagnostic and Production Animal Medicine, College of Veterinary Medicine, Iowa State University, Ames, Iowa, USA

## Abstract

A high-passage rabbit kidney RK-13 cell line (HP-RK-13[KY], originally derived from the ATCC CCL-37 cell line) used in certain laboratories worldwide is contaminated with noncytopathic bovine viral diarrhea virus (ncpBVDV). On complete genome sequence analysis, the virus strain was found to belong to BVDV group 1b.

## GENOME ANNOUNCEMENT

Bovine viral diarrhea virus (BVDV) is a nonenveloped, positive-sense, single-stranded RNA virus with a genome size of approximately 12.3 to 12.5 kb. The virus is a member of the genus *Pestivirus* in the family *Flaviviridae*, which also includes border disease virus and classical swine fever virus (1, 2). Two major genotypes of BVDV are recognized (types 1 [BVDV-1] and 2 [BVDV-2]). BVDV-1 strains are genetically subdivided into at least 17 subtypes (a to q) and BVDV-2 strains into four subtypes (a to d) (3–6). In addition, two distinct biotypes within each genotype have been identified: cytopathic viruses (cpBVDV) that cause cytopathic effects in cultured cells and noncytopathic viruses (ncpBVDV) that do not cause cytopathic effects in cultured cells (7–10). It was previously reported that a number of cell lines (e.g., cattle, sheep, goat, deer, bison, rabbit, and domestic cat origins), including the RK-13 cell line (CCL-37; American Type Culture Collection [ATCC], Manassas, VA) are persistently infected with ncpBVDV, resulting from the use of BVDV-contaminated fetal bovine serum in cell culture medium (11–15). Many laboratories use the RK-13 cell line from the ATCC or its derivatives for research and laboratory confirmation of various viral agents. Our laboratory has been using high-passage RK-13 cells (P399-409; HP-RK-13 [KY]) for routine laboratory diagnostic investigation for >50 years (16). In this study, we determined the complete genome sequence of ncpBVDV present in the HP-RK-13 [KY] cells (P404) using next-generation sequencing (NGS) technology on an Illumina MiSeq platform, according to previously established procedures (17). The sequences were mapped to all known BVDVs, and mapped read sets were used for *de novo* assembly using ABySS version 1.3.7 (BC Cancer Agency, Vancouver, Canada) and Geneious 7.0.6 software (Biomatters Ltd., Auckland, New Zealand).

The complete genome of ncpBVDV contaminating the HP-RK-13 [KY] cell line (ncpBVDV HP-RK-13 [KY] strain) is composed of 12,271 nucleotides (nt) and contains a 5′ untranslated region (UTR) (386 nt), a single open reading frame (ORF) (11,697 nt [nt 387 to 12083]), and a 3′ UTR (188 nt). The single ORF encodes a 3,898-amino acid polyprotein, which is predicted to be cleaved into 12 proteins. The ncpBVDV HP-RK13 [KY] strain had 85.2% to 99.7% identity with 11 strains of BVDV-1b and 68.6% to 70.9% identity with eight strains of BVDV-2 at the whole-genome level. Interestingly, the ncpBVDV HP-RK-13 [KY] strain is very closely related to the previously described ncpBVDV present in RK-13 cells reported from Japan (RK13/E^-^ strain [GenBank accession no. JX419397.1]; 12,064 nt, 99.7% identity) (18). The ncpBVDV HP-RK-13 [KY] strain had several nucleotide insertions and deletions compared to several of the other BVDV-1b strains. However, the specific insertion(s) and/or deletion(s) that are responsible for the establishment of persistent infection in the HP-RK-13 [KY] cell line have not been determined. Phylogenetic analysis of the complete genome sequence of the virus ncpBVDV HP-RK-13 [KY] established that it is of the BVDV-1b genotype.

### Nucleotide sequence accession number.

The complete genomic sequence of ncpBVDV KY-HP-RK-13 strain has been submitted to GenBank under accession no. KT355592.
